# Pancreatic ductal adenocarcinoma: the latest on diagnosis, molecular profiling, and systemic treatments

**DOI:** 10.3389/fonc.2024.1386699

**Published:** 2024-07-01

**Authors:** Doaa Bugazia, Ebtesam Al-Najjar, Abdullah Esmail, Saifudeen Abdelrahim, Karen Abboud, Adham Abdelrahim, Godsfavour Umoru, Hashem A. Rayyan, Ala Abudayyeh, Ala-Eddin Al Moustafa, Maen Abdelrahim

**Affiliations:** ^1^ Department of Medicine, Massachusetts General Hospital, Boston, MA, United States; ^2^ Section of GI Oncology, Department of Medical Oncology, Houston Methodist Cancer Center, Houston, TX, United States; ^3^ Challenge Early College HS, Houston Community College, Houston, TX, United States; ^4^ Department of Pharmacy, Houston Methodist Hospital, Houston, TX, United States; ^5^ Endoprothic Center, Hochwald Hospital, Frankfurt, Germany; ^6^ Department of Medicine, Faculty of Medicine, The University of Jordan, Amman, Jordan; ^7^ Section of Nephrology, Division of Internal Medicine, The University of Texas MD Anderson Cancer Center, Houston, TX, United States; ^8^ College of Medicine, Qatar University, Doha, Qatar; ^9^ Department of Medicine, Weill Cornell Medical College, New York, NY, United States

**Keywords:** pancreatic ductal adenocarcinoma, molecular profiling, pancreatic neoplasm, computed tomography (CT), magnetic resonance imaging (MRI), pancreatic cancer treatments, pancreatic cancer novel therapy

## Abstract

Pancreatic ductal adenocarcinoma (PDAC) is currently the fourth leading cause of death in the United States and is expected to be ranked second in the next 10 years due to poor prognosis and a rising incidence. Distant metastatic PDAC is associated with the worst prognosis among the different phases of PDAC. The diagnostic options for PDAC are convenient and available for staging, tumor response evaluation, and management of resectable or borderline resectable PDAC. However, imaging is crucial in PDAC diagnosis, monitoring, resectability appraisal, and response evaluation. The advancement of medical technologies is evolving, hence the use of imaging in PDAC treatment options has grown as well as the utilization of ctDNA as a tumor marker. Treatment options for metastatic PDAC are minimal with the primary goal of therapy limited to symptom relief or palliation, especially in patients with low functional capacity at the point of diagnosis. Molecular profiling has shown promising potential solutions that would push the treatment boundaries for patients with PDAC. In this review, we will discuss the latest updates from evidence-based guidelines regarding diagnosis, therapy response evaluation, prognosis, and surveillance, as well as illustrating novel therapies that have been recently investigated for PDAC, in addition to discussing the molecular profiling advances in PDAC.

## 1 Introduction

The latest data from the Centers for Disease Control and Prevention (CDC) shows that PDAC claimed fourth in leading causes of cancer deaths in the United States and is expected to be the second leading cause of cancer deaths by soon coming years (1, 2). This devastating disease has a poor prognosis and is rising in incidence. Late presentation of PDAC, and lack of early specific symptoms, result in locally advanced or metastatic disease at the time of the diagnosis, making PDAC one of the worst malignancies, resulting in a low survival rate for the majority of patients (3). Patients with PDAC have a 5-year survival rate of 10% (4). In addition, early diagnosis of the disease is challenging because of the deep location of the pancreas, and its aggressive nature, which translates into late disease discovery at diagnosis. Furthermore, there is an urgent need to find more therapeutic options for this malignancy, and there is a need for new screening or detection strategies to ultimately improve survival outcomes for patients with PDAC. A greater understanding of PDAC pathophysiology has come to light in the last 10 years (5, 6). However, given that PDAC mostly results from frequently mutated somatic cell genes, and less commonly from germline cell mutations, few breakthroughs in reducing the incidence and mortality of the disease have been achieved despite new discoveries of screening tests and emerging therapeutic tools (7, 8).

## 2 Diagnosis methods for pancreatic ductal adenocarcinoma

**Figure 1:**
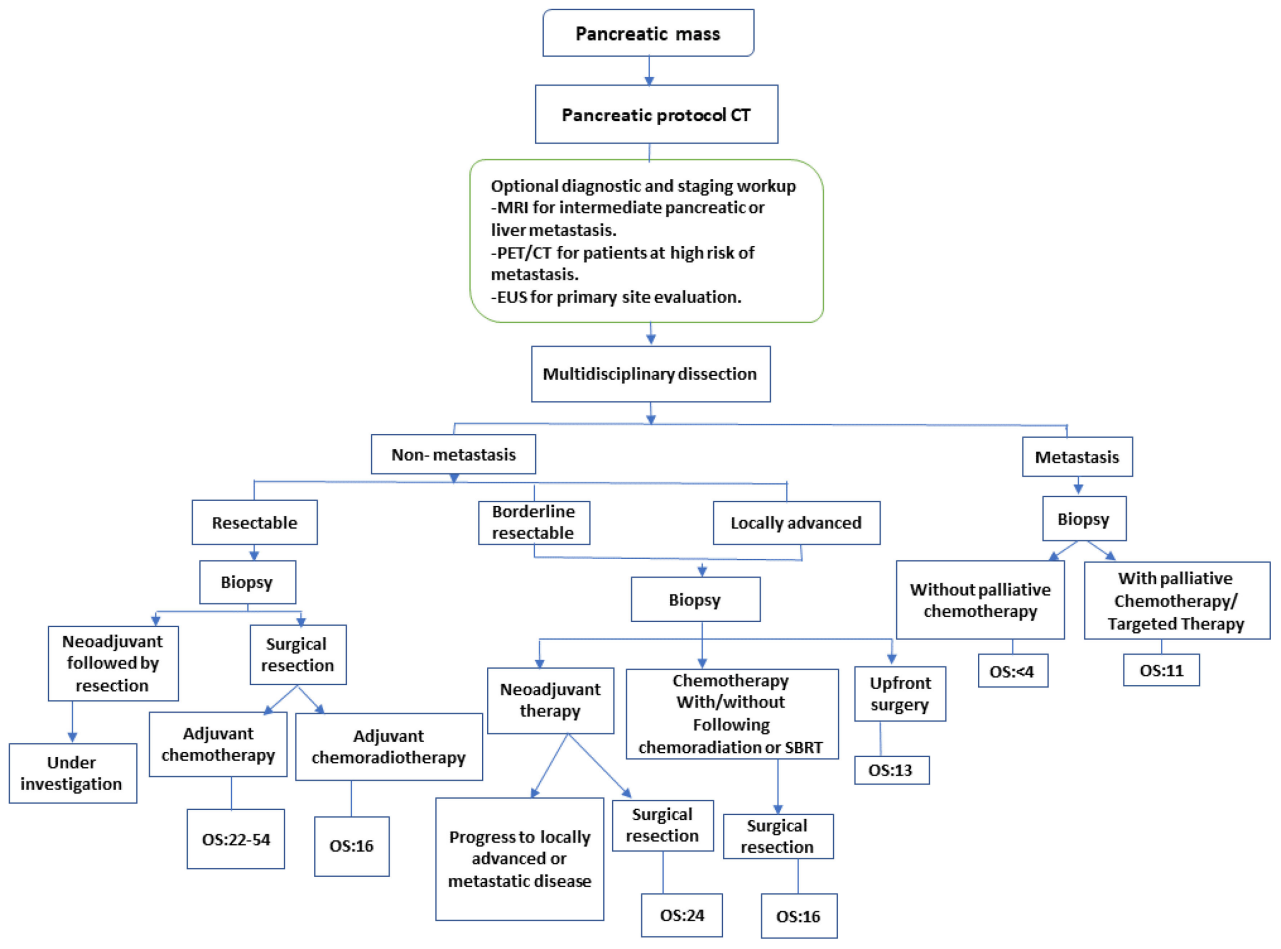
Treatment and staging strategy of pancreatic cancer. CT, Computed tomography; EUS, endoscopic ultrasound; MRI, magnetic resonance imaging; PET, positron emission tomography; SBRT, stereotactic body radiation therapy (9). NCCN guidelines recommend that biopsy for proof of malignancy is not required prior to surgical resection and should not delay surgical resection in patients with high clinical suspicion of pancreatic cancer.

The current diagnostic and therapeutic options for PDAC are convenient for the purpose of staging, tumor response evaluation, and management of borderline resectable or resectable PDAC as shown in **Figure 1**. Additional innovations in imaging tools such as magnetic resonance cholangiopancreatography (MRCP), endoscopic retrograde cholangiopancreatography (ERCP) computed tomography (CT), and ultrasound endoscopy (EUS) even aid in early diagnosis, including carcinoma *in situ* or stage 0 PDAC. MRCP, in particular, can detect abnormalities in pancreatic ducts, while EUS has the potential to detect some areas that might help with diagnosis known as infiltrative distinct hypoechoic appearance that could be associated with dilatation in the pancreatic duct or bile duct in stage 0 PDAC. In the pathological aspect, ultrasound endoscopy fine-needle aspiration (EUS-FNA) and ERCP with the serial pancreatic-juice aspiration cytologic examination (SPACE) test might have the wanted effectiveness in the definitive diagnosis of stage 0 PDAC. The efficiency of early-stage diagnosis of PDAC increases when EUS and MRCP are proactively performed on people at a higher risk of PDAC development. However, in the instances of pancreatic ductal stenosis, caliber change, and pancreatic branch ductal dilation, SPACE is considered ideal for pathological diagnosis. Particularly, SPACE has been found useful for diagnosing early-stage PDAC that is not visible with multiple imaging techniques. Additionally, early-stage PDAC, including atypical epithelium cells of the pancreatic duct can be detected by probe-based confocal laser endomicroscopy (pCLE) with ERCP.

The current utilization of EUS-FNA and SPACE facilitates accurate pathological diagnosis of PDAC at an early stage, though innovation strives to improve diagnostic outcomes. In the future, improved detection on imaging evaluation, of early PDAC, paired with the possibility of definite diagnosis by pathological assessment with EUS and/or SPACE will lead to an increased number of early diagnoses of PDAC. Those early diagnoses will pave the way for curative treatments and improved prognoses in patients with PDAC.

The rising incidence and mortality rates for PDAC, and its management involves a specialized integrated team, including surgical resection, diagnostic scans, interventional tools such as endoscopy, radiation oncology, medical oncology, pharmacy, pathology, geriatric medicine, and palliative care personnel. The adequate reassessment of PDAC tumor and staging post-neoadjuvant therapy allows for an integrated team to select the most appropriate treatment approach for the patient. Additionally, considering lymphovascular and extranodal invasion is crucial in determining the prognosis and proper management of each patient’s treatment. This coincides with several studies that have demonstrated a positive correlation between lymphovascular invasion and poor prognosis in PDAC patients (10–12). Therefore, it is crucial that any signs of lymph node (LN) metastasis should be identified and included when determining patients’ prognosis and management (10–12).

On the other aspect, to establish more advanced diagnostic tools for early PDAC, the development of liquid biopsy research will be crucial and essential to compensate for the limitations of scan techniques. Recent advancements in liquid biopsies have been remarkable, and novel applications such as circulating tumor cells (CTCs), circulating tumor DNA (ctDNA), and methylated circulating-free DNA (cfDNA) have the potential to serve as prognostic and predictive markers. Specific to PDAC, it is difficult, at this stage, to utilize liquid biopsy for early disease diagnosis, although, the four major genes including the TP53, KRAS, CDKN2A4, and SMAD4 mutations that occur in PDAC are useful for monitoring the treatment response and course of pancreatic cancer. For example, a blood test diagnosis of early-stage pancreatic cancer examines the optimal combination of the four major genes and the discovery of new genes commonly expressed in the early stages of pancreatic cancer. Currently, there are two, inferred, key focal points in liquid biopsy research. Initially, several reports of molecular profiling mainly use KRAS, but investigations are ongoing for new markers indicative of early-stage PDAC. Molecular biological analysis techniques such as digital polymerase chain reaction (PCR) can enhance the accuracy of imaging and liquid biopsy findings and facilitate the early detection of PDAC.

### 2.1 PDAC imaging tools

#### 2.1.1 Artificial Intelligence

In a recent study large -scale pancreatic cancer detection via non-contract CTscan and deep learning was done by Kai Ca et al, who mentioned that a team of researchers has developed an innovative solution named Pancreatic Cancer Detection with Artificial Intelligence (PANDA). This tool effectively identifies and classifies pancreatic lesions using a non-contrast CT scan. PANDA, after training on data from 3,208 patients from one center, proved to be highly effective. In a large test across 10 centers with 6,239 patients, it achieved an impressive score on the receiving operating characteristic curve, ranging from 98.6% to 99.6% for detecting lesions. Compared to the average radiologist, PANDA showed significant improvement by 34.1% in sensitivity and 6.3% in specificity for identifying PDAC. Furthermore, in a real-world test with 20,530 patients, PANDA demonstrated a sensitivity of 92.9% and a specificity of 99.9% in detecting lesions across various scenarios.

#### 2.1.2 Photon-counting detector CT

A retrospective study that analyzed the detection of PDAC by virtual monoenergetic images(VMI) on a novel photon-counting detector CT (PCD-CT) comparison to energy-integrating CT(EID-CT), showed that PCD-CT VMI provided notably better visibility of PDAC in both arterial and portal venous contrast phase compared to EID-CT, as a confirmed by thorough quantitative and qualitative assessments. This enhanced visibility is crucial for early tumor detection in clinical practice. Notably, tumor delineation was superior during the portal venous contrast phase compared to the arterial contrast phase.

#### 2.1.3 Fibroblast activation protein inhibitor(FAPI)_04 PET/CT:

A study investigated FAPI_04 PET-CT to detect sixty-tow patients with PDAC and compared the result with those undergoing ^18^F-FDG PET/CT. The study notably that FAPI_04 PET/CT outperformed ^18^F-FDG PET/CT in detecting primary tumor, lymph node metastasis, distant metastasis, and accurately staging PDAC according to TNM classification.

#### 2.1.4 Spectral imaging mode

Spectral CT has the potential to improve iodine specificity. Intuitively, this could improve the visualization of PDAC, and some studies have proposed a method to differentiate chronic pancreatitis from PDAC, which is a well-known problem. In conjunction with dual-energy CT, recently discovered photon counting detectors appear to be able to overcome the spatial and contrast resolution of conventional CT images. For example, Yin et al. reported their investigation of dual-energy MDCT in spectral imaging in the differential diagnosis of PDAC and chronic mass-forming chronic pancreatitis (CMFP) during the arterial and the pancreatic phase. This study showed that individual patient CNR-optimized energy level scans and the NIC might be used to enhance the sensitivity and specificity for identifying CMFP from PDAC by use of dual-energy MDCT in spectral scans with fast tube voltage switching.

#### 2.1.5 CT scans

The CT scans of PDAC may illuminate findings such as hypoattenuating masses, abrupt ductal cut-off at the site of the masses, double duct signs (a combined dilatation of the common bile duct and pancreatic duct), poor enhancements of pancreatic, venous phases comparative to normal pancreatic parenchyma, and the tendency of isoattenuation to normal pancreatic parenchyma in delayed phases. Meta-analysis of trials has shown that CT scans have a sensitivity of 89–91% and a specificity of 85–90% in the detection of PDAC (13–15). However, CT scanning has demonstrated poor diagnostic accuracy for small hepatic, peritoneal, or lymphatic tissue metastasis (16–18).

According to the European Society for Medical Oncology (ESMO) and the National Comprehensive Cancer Network (NCCN) guidelines (19, 20), CT scanning is the currently recommended as the primary imaging modality when evaluating or assessing patients for surgical resectability. CT scans can show great spatial and temporal resolution as well as a wide anatomic overview. However, CT images should conform to the pancreatic protocol that is recommended for precise evaluation of tumor staging (21). Although PDAC can advance and spread rapidly, an imaging examination should be conducted within a month following final therapy (22). CT scan utilization for therapeutic decision-making consists of a thin (preferably submillimeter) continuous slice with 3 mm reconstruction and maximum intensity projection, or 3-dimension (3D) volumetric thick section scans for blood vessels assessment and multiplanar reformation encompassing the coronal plane. Both the pancreatic level (40–50 seconds after intravenous contrast injection) and the venous phase (65–70 seconds) should be involved in evaluating pancreatic masses or lesions and encompassing vasculature (13, 20). PDAC is typically visible in the pancreatic location as a mass lesion with hyperenhancement relative to the neighboring parenchyma. It may produce pancreatic/bile duct occlusion and upstream dilatation, direct invasion of nearby organs, abutment or encasement of adjacent arterial systems, and expansion of regional lymph nodes. The most common anatomical metastatic destinations are the liver, peritoneum and distant lymph nodes. Around 5% of PDAC may exhibit isoattenuation in both venous and parenchymal levels (14, 15).

#### 2.1.6 MRI

In a meta-analysis of trials focused on PDAC diagnosis, MRI scans demonstrated a sensitivity of 84–93% and a specificity of 82–89% (16–18, 23), which coincides with additional MRI with diffusion-weighted imaging (DWI) data for hepatic masses seen in an additional study (24). Results indicated that MRI with DWI may be useful in the recognition of hepatic metastases in around 1.5–2.3% of individuals with no visible mass on CT, and about 10.5–13.6% of patients with ambiguous liver lesions who meet the criteria for upfront surgery (24). Though other research indicates that MRI with tissues of hepatobiliary contrast using gadoxetic acid, in particular, is more sensitive than CT (85% vs. 69%) and is more valid for distinguishing between metastases and hepatic microabscesses (17, 25).

Regardless, because the majority of PDAC exhibits limited diffusion, DWI maybe the best assistance in the detection of this malignancy (26, 27). The addition of an extra MRI scan also may modify the findings of resectability assessments in a substantial number of patients (14.4%) due to greater sensitivity for liver metastases (28). Though careful observations will still be required, especially regarding DWI for pancreatitis, which presents with limited diffusion, and is difficult to identify or distinguish from PDAC. Additionally, DWI has decreased spatial resolution and is susceptible to artifacts induced by intestinal gas or movements (29). Regarding PDAC assessment, MRI must incorporate these sequences: T1-weighted in-and-out of level gradient-echo; T2-weighted fast spin-echo; T2-weighted fat-suppressed fast spin-echo; DWI; 3D T1-weighted fat-suppressed gradient-echo dynamic images. As well as, bringing in the additions of precontract, pancreatic, venous, and equilibrium phases; and T2-weighted MRCP sequences (19). PDAC is typically hypo-intensified in precontract T1-weighted images and has a varied signal strength, with or without fat suppression in T2-weighted images (30). Additionally, the available range of various sequences and MRI’s superior soft-tissue contrast have the potential to aid in the identification and characterization of tiny, subtle, cystic, or potentially attenuating pancreatic and liver lesions. On T2-weighted imaging or MRCP, PDAC frequently shows dilatation of the pancreatic duct or cutoff.

MRCP can non-invasively show and demonstrate pancreatic and bile duct abnormalities, including anatomic variances and obstructive dilatation. With these benefits, MRI is a useful imaging modality for ambiguous pancreatic lesions (particularly tiny or attenuating tumors) or very small liver masses or lesions.

Of note, MRI has some limitations, including reduced spatial resolution, susceptibility to artifacts’ motion, and extremely limited multiplanar reformation capabilities. In spite of these limitations, MRI has demonstrated diagnostic performance comparable to CT.

#### 2.1.7 PET

PET/CT scans the body as a whole and are, particularly, useful for detecting distant metastasis, though, might also assist with lymph node staging (31, 32). The NCCN guidelines recommend that PET/CT must not be used in place of pancreatic CT or MRI. However, PET/CT maybe utilized as adjunct imaging in patients at high risk of metastatic diseases, such as those with severely elevated CA19–9 levels, borderline resectable disease, large primary mass, large regional lymphatic nodes, and or characteristic presentation (19). For the diagnosis and detection of PDAC, 18FDGPET/CT has a sensitivity of 89–91% and a specificity of 70–72% (18, 23). The most often utilized radiotracer in PET scans is 18Fluorine-2-fluoro-2-deoxy-D-glucose (18FDG). As a glucose analog, 18FDG, enables *in vivo* scanning of glycolytic activity, which is commonly enhanced in solid tumors and PDAC.

The possible benefits of 18FDGPET or 18FDG-PET/CT compared to pancreatic CT in the diagnosis of PDAC are still questionable (18, 31–33). Both hypoxic microenvironment and the KRAS mutation, which is seen in more than 90% of PDAC patients, promote 18FDG absorption via upregulation of the expression of HK2 and GLUT1 (34). However, because localized pancreatitis can cause increased 18FDG absorption, distinguishing between PDAC and focal pancreatitis can be challenging (34).

#### 2.1.8 Endoscopic ultrasound

For the detection of PDAC, EUS has a sensitivity of 89–91% and a specificity of 81–86% (18, 23). EUS has an advantage over CT scans due to its superior spatial resolution, which may be utilized to provide additional details for pancreatic malignancies when the pancreatic lesion is ambiguous on CT or when there is a dubious blood vessel or lymph node involvement (35, 36). However, the use of EUS as a regular imaging technique for resectability assessments is still debatable. The ESMO recommendations include EUS as a regular imaging technique; however, the NCCN guidelines do not. The primary purpose of EUS is to diagnose pathology by way of FNA. Furthermore, the diagnostic potential of EUS is restricted because of operator reliance and anatomic variability of the superior mesenteric and celiac arteries.

### 2.2 Tumor markers for PDAC

#### 2.2.1 CA19–9

Tumor cells that have documented changes in carbohydrate structure correlate with different types of cancer (37). CA19–9 is the most broadly investigated and validated biomarker regarding diagnostic, prognostic, and surveillance capacity (38). Hence, it is the only FDA-approved biomarker tool for monitoring and diagnosis of PDAC (39). A thorough meta-analysis of 2283 individuals demonstrated a median specificity of 82% and a median sensitivity of 79% for the diagnosis of PDAC in symptomatic patients (39). These observations have been confirmed in subsequent investigations on the use of CA19–9 in the diagnosis of PDAC (40–42). However, some limitations of CA 19–9 reduce its utility as a biomarker. To begin with, due to Lewis antigen dependency, around 5–10% of the Caucasian population has significantly decreased CA19–9 production (39). As a result, current research is focusing on defining distinct kinds of CA19–9 secretions based on the patient’s Lewis antigen status and other characteristics (43, 44). Furthermore, nonmalignant conditions such as liver cirrhosis, obstructive jaundice, chronic pancreatitis, and cholangitis can also elevate CA19–9 (45) (39). Additionally, the limited positive predictive value of CA19–9 limits its use as a screening tool in larger populations (46). Other carbohydrate antigens have been studied for their diagnostic utility in early detection of PDAC. Prior studies have connected CA125, CA19–9, CA242, and CA50 to PDAC (47–49). However, none of these carbohydrate antigen indicators show diagnostic potential unless they are combined with CA19–9. This combination may assist in distinguishing between benign and malignant pancreatic tumors (50).

Using biomarkers to improve postoperative surveillance might be extremely beneficial for patients with PDAC. Clinically, the most common biomarker for PDAC is CA19–9 (51). According to Li et al., early identification of CA19–9 as a marker for PDAC recurrence following resection can enhance patient prognosis in terms of disease-free survival by allowing them to begin salvage chemo-therapy earlier (52). Despite having a maximum specificity of 89% and a maximum sensitivity of 89% (53), several studies have shown that elevated CA19–9 levels often precede the radiological evidence in detecting recurrence for up to 3–6 months. However, these studies focused on estimating prognosis rather than identifying biomarkers for postoperative surveillance. In a scientific research, Li et al. reported that CA19–9 elevations of > 210 U/mL before surgery and elevations of > 37 U/mL after surgery were shown to be independently related to early recurrence of PDAC. To summarize, the CA19–9 biomarker is the most readily tracked for postoperative PDAC monitoring to date.

#### 2.2.2 CEA

Studies on postoperative surveillance of PDAC have indicated that CEA is inferior to CA19–9 in early diagnosis of PDAC, with a specificity of 65% and a sensitivity of 50% (54). In early diagnosis of the PDAC, CEA’s value elevation has poorer sensitivity and specificity than CA19–9 (40, 55). As a result, CEA appears to have a higher predictive value when paired with CA19–9, especially in advanced PDAC (53).

#### 2.2.3 CtDNA

The CTCs are tumor cells shed from primary or metastatic sites that reach the peripheral circulation (56). For this reason, CTCs are currently studied for their use as biomarkers. Favorable studies demonstrate the capacity of digital PCR and next-generation sequencing (NGS)-based technologies to detect ctDNA. While the utility of ctDNA has been validated in many cancers such as colorectal, lung and thyroid cancers, it has not yet become part of routine clinical practice in PDAC. CtDNA can be leveraged to detect common mutations in genes of interest such BRAF, EGFR, PIK3CA, KRAS, P53, etc. (57–62). Moreover, ctDNA has recently been utilized in clinical trials because it allows monitoring of tumor response to targeted therapy, tracking the development of resistance, and even detecting minimal residual disease (62–66). For these reasons, ctDNA may potentially be used to predict disease status prior to imaging. For example, the presence of ctDNA after surgery in early-stage PDAC is associated with reduced recurrence-free survival during the monitoring phase. However, more research is needed to determine whether ctDNA can be used as a biomarker for PDAC, MRD and surveillance (67–69). According to several recent studies, the analysis of CTCs, cfDNA or RNA, exosomes and secretomes are promising approaches to be utilized for molecular profiling. Furthermore, high blood levels of cfDNA, and CTCs have been detected in late-stage PDAC. Therefore, analyzing cfDNA and CTCs is non-invasive and able to provide useful information for approaching or managing the PDAC (70–74).

## 3 Treatment options for PDAC

Treatment outcomes and median overall survival depend, primarily, on the initial staging of PDAC. The only curative therapy option for patient’s diagnosed with PDAC is surgical excision (75). Unfortunately, by the time of diagnosis, only 10–20% of patients meet resection criteria for curative treatment, and more than half of patients have metastatic disease upon presentation (76). At the present, PDAC treatment differs based on the clinical and anatomical staging (77). In 2017, the NCCN published guidelines concerning eligibility criteria for surgical curative resection; the main objective being to include as many patients as possible under the sole surgical curative option (20). In non-metastatic PDAC, imaging modalities may be used to categorize the patient as resectable, borderline resectable, or locally advanced (**Figure 1**), depending on the extent of the disease to surrounding anatomical arterial (superior mesenteric artery, common hepatic artery, and celiac axis) and anatomical venous (superior mesenteric vein or portal vein) structures, as well as other nearby organs and lymph nodes (78, 79). Several studies are currently underway to identify treatment strategies tailored to each patient’s unique molecular pathology of PDAC (80–82).

### 3.1 Resectable PDAC

Resectability criteria based on the recommendations of the NCCN, and the Consensus (Americas Hepato-Pancreato-Biliary Association AHPBA/Society of Surgical Oncology SSO/Society for Surgery of the Alimentary Tract SSAT) include noninvolvement of the superior mesenteric artery (SMA), celiac artery (CA), and superior mesenteric vein (SMV). Borderline resectable PDAC for SMA contact of less than or equal to 180°, contact with the common hepatic artery (CHA) without extension to CA or less than or equal to 180° contact, or more than or equal to 180° with the out involvement of the aorta. Borderline resectability also allows for more than 180° contact or less than or equal to 180° contact with contour irregularity with short anatomical segment involvement besides is suitable for proximal and distal vessel’s reconstruction. The locally advanced PDAC is classified as distant anatomical metastasis, >180° contact with the SMA or CA, contact with 1^st^ jejunal branch of the SMA, involvement of the aorta, and contact with the 1^st^ draining jejunal branch of SMV or long segment involvement of the SMV with difficult reconstruction (83). Despite resectability, many patients experience local recurrence or metastatic disease. This is hypothesized to occur due to hidden or non-evident micrometastasis (84–86). Considering this fact, medical researchers are conducting investigations into the effect of adjuvant chemotherapy in patients with resectable PDAC. CONKO-001 was a randomized clinical trial that evaluated gemcitabine’s efficacy and safety in the adjuvant setting for PDAC. The researchers wanted to explore the toxicity and efficacy of gemcitabine in cases with resected PDAC (87, 88). Overall, 354 patients were randomly assigned 1:1 to either surgery followed by six months of gemcitabine therapy sessions (treatment group) or just surgical resection (control group). They reported that the treatment group had a longer median overall survival (OS) in comparison with the control (22.8 months vs. 20.2 months; HR (Hazard Ratio), 0.76; p, 0.01). In addition, the therapy group had a superior progression-free survival (PFS) (PFS, 13.4 months vs. 6.7 months; HR, 0.55; p 0.001). Gemcitabine is listed as a category 1 option for resectable PDAC.

Conversely, fluoropyrimidines-based therapy, in particular fluorouracil with leucovorin (5-FU+LV), shows to have a comparable OS rate to gemcitabine (based on the randomized European multicenter, Study Group for PDAC (ESPAC)-III study findings) (89). The median OS for 5-FU/LV was reported to be 23 months (95% CI, 21.1–25.0) vs 23.6 months with gemcitabine (95% CI, 21.4–26.4) without statistical difference (HR, 0.94; 95% CI, 0.81–1.08; p, 0.39). Given that 5-FU/LV is also listed as a category 1 recommendation in NCCN guidelines, it is worth noting that ESPAC-III demonstrated the significance of completing the adjuvant chemotherapeutics instead of just relying on the immediate initiation of chemotherapy following surgery (90). Additionally, according to a subgroup analysis, patients who finished all six scheduled cycles of therapy had a significantly higher OS than participants who did not 14.6 months (95% CI, 12.5–16.9)] versus [28.0 months (95% CI, 26.1–30.9), respectively (HR, 0.516; 95% CI, 0.44–0.60; p, 0.001) (91). On the other hand, chemotherapy administered within two months post-surgery had no effect on the patient survival rate when directly compared to those who received and completed chemotherapy outside an 8 weeks post-op window (total OS of 22.6 months (95% CI, 21.3–25.5) vs 24.2 months (95% CI, 22.3–26.4), in each single case respectively (HR, 0.946; 95% CI, 0.82 to 1.09; p, 0.42). Although, the completion of this entire intervention of adjuvant chemotherapy is an important goal, it may not be feasible in all patients due to major complications and comorbidities, nutritional status, and functional abilities prior and after surgery.

Following the success of both fluoropyrimidine-based therapy and gemcitabine in the adjuvant setting, it was hypothesized that combination treatment may lead to additive benefit. The phase III ESPAC-4 study investigated the efficacy of adjuvant oral capecitabine paired with the use of gemcitabine as a chemotherapy-based regimen. With the median follow-up of 43.2 months, 730 patients were randomly assigned to receive gemcitabine coupled with capecitabine versus gemcitabine alone (90). The median OS with gemcitabine alone was 25.5 months vs. 28 months in the combination group (HR, 0.82; 95% CI, 0.68–0.98; p, 0.032). Additionally, compared to gemcitabine monotherapy, the combination group had a considerably greater 5-year survival rate (28.8% vs. 16.3%). These data show that combining capecitabine with gemcitabine is preferable to using gemcitabine alone and is also listed as a category 1 recommendation by NCCN guidelines. Efficacy of this combination has been attributed to synergistic inhibition of DNA thymidylate by capecitabine and gemcitabine (92).

In another open-label, multicenter phase III study called APACT, the effectiveness of adjuvant gemcitabine-nab-Paclitaxel (GnP) compared to gemcitabine alone for resectable PDAC was assessed. Although GnP resulted in a greater median OS (40.4% vs 36.2%), the combination did not achieve the same degree of clinical significance when it came to disease-free survival (DFS), in comparison to gemcitabine alone (19.4 months vs 18.8 months; p = 0.1824) (93).

Additionally, the PRODIGE-24 multicenter, randomized trial was conducted to examine whether a modified regimen of folinic acid, oxaliplatin, fluorouracil, and irinotecan (mFOLFIRINOX), was more effective than single-agent gemcitabine in the adjuvant setting for resectable PDAC (94). In this study, 493 patients were randomly assigned to receive either mFOLFIRINOX or gemcitabine, with a median follow-up of 33.6 months. When compared to gemcitabine monotherapy, mFOLFIRINOX provided a higher median OS of 54.4 months against 35.0 months (HR, 0.64; 95% CI, 0.48–0.86; p = 0.003), as well as a higher PFS (21.6 months vs 12.8, respectively; HR, 0.58; 95% CI, 0.46–0.73; p = 0.001). mFOLFIRINOX was linked to considerably more adverse events (grade 3 to 4) compared to gemcitabine (75.9% vs. 52.9%); however, all events were reversible, with the exception of oxaliplatin-related neurotoxicity, which persisted in two cases in the mFOLFIRINOX group. It is important to highlight that growth factor support should be considered for patients on mFOLFIRINOX as the regimen carries an intermediate risk for febrile neutropenia.

Notably, strong evidence for chemoradiotherapy in resectable PDAC has not been shown yet. The ESPAC-1 multicenter, randomized trial allocated 145 patients to undergo chemoradiotherapy (alone or in conjunction with adjuvant chemotherapy) versus 144 patients who did not receive chemoradiotherapy (either chemotherapy alone or none) (89). The median follow-up for this study was 47 months with a median OS of 21.6 months (95% CI, 16.5–22.7) for chemotherapy. Based on the results of this trial, chemotherapy had more favorable outcomes when compared to chemoradiotherapy [median OS of 15.9 months (95% CI, 13.7–19.9)].

It is important to note that neoadjuvant treatment (NAT) is a controversial issue that is continuously being researched in the setting of resectable PDAC. In theory, NAT could downsize the tumor, improve the likelihood of achieving free margin resection (R0), and eliminate nonvisible microscopic metastasis (95). However, recent studies have revealed that delaying surgical resection for NAT may endanger both the OS and therapeutic outcomes due to potential local disease metastasis and NAT’s adverse events, as a result of which individuals may be unable to undergo surgical resection in the future (96). In participants with resectable disease, ongoing trials are looking into the importance of upfront NAT. The S1505 SWOG trial, a randomized phase II clinical study, investigated the impact of 3 months pre-operative and 3 months postoperative therapy mFOLFIRINOX vs GnP as a NAT for patients with resectable PDAC (97, 98). The majority (75%) of patients were able to complete NAT and undergo surgery; moreover, 85% of patients had negative (R0) margins. Response to NAT was encouraging as the major pathologic response rate was 33%. Nevertheless, the authors advise caution in the selection of patients who are deemed able to tolerate NAT as ~9% of patients were not able to undergo surgery due to NAT toxicity. Completion of adjuvant therapy was also suboptimal at 63%. Further insight into OS data will be available on longer follow-up. Ultimately, initial results from SWOG 1505 showed that both regimens can be feasible and tolerable when delivered prior to surgery, but the subset of patients able to tolerate and complete this approach needs to be prospectively identified (97). The Alliance A02186 trial will compare perioperative FOLFIRINOX (8 cycles in the neoadjuvant setting and 4 cycles in the adjuvant setting) to 12 cycles of adjuvant FOLFIRINOX (NCT04340141). In the setting of resectable PDAC, both the American Society of Clinical Oncology (ASCO) and the NCCN advocate for upfront resection surgery preceding 6 months duration of adjuvant chemotherapy. However, neither guideline recommends NAT unless the patient is in a high-risk population (individuals with radiological features that raise suspicion of extra-pancreatic illnesses but do not lead to a diagnosis, significantly elevated CA19–9 values, large primary tumors, large regional lymph nodes, significant weight loss, or excruciating pain) (19, 99). According to the most recent NCCN guidelines, the preferred options are gemcitabine monotherapy, gemcitabine with capecitabine, or 5-FU/leucovorin. Furthermore, a meta-analysis of randomized clinical trials has shown that there is a better OS rate when patients with resectable pancreatic cancer are treated with neoadjuvant chemotherapy than surgery as a first approach; moreover, neoadjuvant chemotherapy increased disease-free survival (DFS) when compared to surgery as a first-line approach (100).

### 3.2 PDAC with borderline resectability/local advancement

Currently, NAT is considered crucial in borderline resectable PDAC (**Figure 1**). In addition to its tumor-shrinkage effect allowing for better surgical excision, NAT is associated with fewer complications and a higher likelihood of achieving free margin resection (R0). NAT also lowers the extent of potential involvement of lymph nodes and reduces early undetectable microscopic metastasis all of which improve OS and therapeutic outcome (54, 95, 101, 102).

NAT’s involvement in PDAC therapy has lately been studied in several clinical studies, notably with the announcement of the revised surgical consensus in 2009. A meta-analysis including 96 papers comprising 5520 participants analyzed the role of NAT in resectable, locally advanced, and borderline resectable diseases (103). NAT has an excellent outcome in resectable borderline and locally advanced tumors (**Figure 1**), as observed by 70% and 84% resection rates in borderline resectable diseases as well as 32% and 82% for locally advanced diseases, respectively. Nevertheless, since obtaining R0 resection has been determined to be an independent predictive factor for both survival and disease recurrence (104, 105), it is important to note that the studies included in this meta-analysis were heterogeneous and lacked standardized individual data. Ongoing research is examining whether NAT chemotherapeutic regimens are appropriate for locally advanced PDAC, with the majority of studies recommending first-line therapies, using FOLFIRINOX or GnP. A systematic meta-analysis found that individuals treated with FOLFIRINOX had superior median OS and PFS of 24.2 months (95% CI, 21.6–26.8) and 15.0 months, respectively (95% CI, 13.8–16.2) (106). It is important to clarify that there have been no studies comparing FOLFIRINOX to GnP as NAT used in the locally advanced or borderline resectable PDAC.

Preoperative chemoradiotherapy (CRT) is effective in borderline PDAC, according to results from the PREOPANC trial. The PREOPANC trial randomly assigned 246 patients to one of two groups: preoperative gemcitabine-based chemoradiotherapy or upfront surgery, with both groups receiving adjuvant gemcitabine after surgery (107, 108) (**Table 1**). Although the difference in median OS was not clinically significant (15.7 months v 14.3 months), the 5-year OS rate showed a clinically relevant improvement of 14% in favor of neoadjuvant gemcitabine-based chemoradiotherapy, and the benefit was consistent across all subgroups, including resectable and borderline resectable disease. ESPAC5 compared 4 different approaches: neoadjuvant FOLFIRINOX, neoadjuvant gemcitabine with capecitabine, chemoradiotherapy, neoadjuvant capecitabine-based chemoradiation, and upfront surgery in borderline resectable PDAC. Following surgical resection, all patients received adjuvant chemotherapy at the physician’s discretion. No difference was observed in the primary endpoint of resection rate between upfront surgery and neoadjuvant treatment; similarly, R0 resection was numerically but not statistically significantly improved with neoadjuvant treatment. On the other hand, the 1-year OS rate, which was a secondary endpoint, was 39% for upfront surgery, 78% for gemcitabine with capecitabine, 84% for FOLFIRINOX, and 60% for capecitabine-based chemoradiotherapy (p=0·0028). ESPAC5 demonstrated that neoadjuvant chemotherapy conferred a better survival advantage over neoadjuvant chemoradiotherapy and upfront surgery (112).

**Table 1:** Pivotal trials that have provided evidence for treatment of resectable, borderline resectable, and metastatic PDAC.

SUMMARY OF PIVOTAL TRIALS PROVIDING EVIDENCE FOR RESECTABLE PANCREATIC ADENOCARCINOMA						
Study	No. of patients	Intervention	Comparator	Median Follow-Up	mPFS	mOS
**CONKO-001 (** 88)	354	Adjuvant Gemcitabine	Surgical resection only	136	13.4 vs 6.7 months; HR, 0.55; p< 0.001.	22.8 vs 20.2 months; HR, 0.76; P, 0.01
**ESPAC-4 (** 90)	730	Adjuvant Gemcitabine + Capecitabine	Adjuvant Gemcitabine	43.2 months	13.9 vs 13.1 months (HR 0.86, P<.001)	28 months vs 25.5 months (HR, 0.82; 95% CI, 0.68–0.98; P, 0.032)
**APACT (** 93)	866	Adjuvant Gemcitabine + nab-paclitaxel	Adjuvant Gemcitabine	63.2	No statistical difference	41.8 vs 37.7 months, HR 0.80, P= 0.0091
**PRODIGE-24 (** 94)	493	Adjuvant mFOLFIRINOX	Adjuvant Gemcitabine	33.6 months	(21.6 months vs 12.8, respectively. HR,0.58; 95% CI, 0.46–0.73; p < 0.001)	54.4 vs 35.0 months (HR, 0.64; 95% CI, 0.48–0.86; p = 0.003)
**PREOPANC (** 108)	246	Neoadjuvant gemcitabine followed by surgery then followed by adjuvant Gemcitabine	Surgical resection followed by adjuvant Gemcitabine	59	–	15.7 months vs 14.3 months; HR, 0.73; 95% CI 0.56–0.96; P, 0.025
SUMMARY OF PIVOTAL TRIALS PROVIDING EVIDENCE FOR METASTATIC PANCREATIC ADENOCARCINOMA						
Study	No. of patients	Intervention	Comparator	Median Follow-Up (months)	mPFS	mOS
**MPACT (** 109)	861	Gemcitabine + nab-paclitaxel	Gemcitabine	9.1 vs. 7.4	5.5 vs 3.7 months; HR, 0.69; P<0.001	8.7 months vs. 6.6 months; HR for death, 0.72; 95% CI, 0.62–0.83; p 0.001
**PRODIGE (** 94)	342	FOLFIRINOX	Gemcitabine	26.6	6.4 vs 3.3 months (HR 0.47, P< 0.001)	11.1 vs 6.8 months (HR 0.57, P< 0.001)
**NAPOLI-1 (** 110)	417	Liposomal Irinotecan (nal-IRI) plus 5FU plus leucovorin (LV)	5FU plus leucovorin (LV)		3.1 vs 1.5 (N/F V FU; HR 0.56, P = .001); 2.7 vs 1.6 (N v FU; HR 0.81, P= 0.1)	6.1 vs 4.2 (N/F v FU; HR 0.67, P= 0.12) 4.9 v 4.2 (N v FU; HR 0.99, P=0.94)
**NAPOLI-3 (** 111)	770	Liposomal Irinotecan (nal-IRI) plus oxaliplatin plus 5FU and leucovorin (LV)	Gemcitabine + nab-paclitaxel	16.1	7.4 vs 5.6 months; HR 0.70 [0.59–0.84]; p = 0.0001	11.1 vs 9.2 months (HR 0.84 [95% CI 0.71–0.99]; p = 0.04)

NCCN guidelines, which are also comparable to ASCO guidelines from 2019, do not recommend upfront resection for borderline resectable or locally advanced illness, and highlight FOLFIRINOX, gemcitabine-nab-paclitaxel, and gemcitabine-cisplatin (especially in individuals with DNA repair mutations) as appropriate therapeutic options (20, 99). CRT is useful as a NAT for patients with poorly controlled intense pain, localized invasions with internal bleeding, worsening performance status, and a progressive local malignancy despite chemotherapy, but no evidence of metastatic disease (20, 99). According to Frassini et al., which reviewed intraperitoneal chemotherapy option for pancreatic cancer patients, hyperthermic intraperitoneal chemotherapy (HIPEC) could be utilized as a preventative approach for peritoneal metastasis in patients with borderline resectable and locally advanced pancreatic cancer. Also, the study highlighted pressurized intraperitoneal aerosol chemotherapy (PIPAC) and normothermic intraperitoneal chemotherapy (NIPEC) as palliative options for patient with unresectable pancreatic cancer, especially in the setting of their encouraging survival rate in comparison to other options reported in literature (113).

### 3.3 Metastatic PDAC

Distant metastasis PDAC has the poorest prognosis among the different stages of PDAC, with only 7% achieving a median one-year survival rate (114). Treatment options for metastatic PDAC are minimal, with the main therapeutic goal being palliation especially in patients with poor performance status and significant comorbidities present at diagnosis. Since the late 1990s, gemcitabine has been used as the first-line treatment regimen for treating metastatic PDAC, according to evidence from published studies demonstrating gemcitabine’s superiority over 5FU in improving overall OS (115, 116) (**Table 1**). During the previous two decades, extensive research focused on enhancing the efficacy and results of gemcitabine by combining it with another cytotoxic medication. Gemcitabine in conjunction with other fluoropyrimidine-based therapy has proved to be more effective than gemcitabine alone (117). Cunningham et al., for example, showed that the combination of gemcitabine and capecitabine (GEM-CAP) exhibited a higher median OS and PFS than gemcitabine (GEM) alone (118). The median OS for GEM–CAP was 7.1 months while GEM had a 6.2-month median OS (HR, 0.86; 95% CI, 0.72–1.02; p 0.08). GEM-CAP had a 1-year OS rate of 24.3%, whereas GEM had a rate of 22%. The PFS in the group of GEM-CAP was 5.3 months versus 3.8 months in the GEM group (HR,0.78; 95% C, 0.66–0.93; p < 0.004). At 12 months, PFS rates for GEM-CAP and GEM were 13.9% and 8.4%, respectively (118). On the flip side, in a phase 3 clinical trial conducted by the National Cancer Institute of Canada (NCIC CTG) called the PA.3 study, erlotinib in combination with gemcitabine, yielded a statistically insignificant improvement in the median OS (6.24 months in the gemcitabine and erlotinib combination group vs 5.91 months in the gemcitabine group; HR, 0.82; 95% CI, 0.69–0.99; p, 0.04) (119, 120). Individuals with greater functional and less pain levels at the point of diagnosis had slightly better outcomes. These data suggest that combining gemcitabine with cytotoxic agents results in minor improvements in comparison with gemcitabine alone. In contrast, the combined regimen of albumin-bound paclitaxel and gemcitabine constituted a paradigm shift in the treatment of advanced PDAC (121). In the phase III MPACT trial, albumin-bound paclitaxel and gemcitabine significantly improved median OS compared to gemcitabine alone in advanced PDAC (8.7 months vs 6.6 months; HR for death, 0.72; 95% CI, 0.62–0.83; p 0.001) (121). Even for those with high-risk traits such as greatly elevated CA19–9 levels, combination therapy improved the OS (HR, 0.612, 95% CI, 0.49–0.76, p < 0.001) (**Table 1**). FOLFIRINOX is still preferred over gemcitabine-based regimens for treatment of advanced PDAC for patients with a decent ECOG performance scale status (PS) owing to the longer median OS and tolerance of adverse outcomes in this population based on the PPRODIGE trial. In the phase III PPRODIGE trial, 342 participants with metastatic PDAC with an ECOG performance rating of 1 or less were either randomized to FOLFIRINOX or gemcitabine for 6 months in 2011 **Table 1**. FOLFIRINOX resulted in 11.8-month of median OS and a 6.6-month of PFS, whereas gemcitabine had a median OS of 6.8 months with a PFS of 3.3 months (HR,0.57; 95% CI, 0.45–0.73; p 0.001) (**Table 1**) (122). Although, FOLFIRINOX was linked to a higher toxicity rate, particularly febrile neutropenia, the FOLFIRINOX group had a better 6-month deterioration of life than the gemcitabine monotherapy group (31% vs 66% respectively) (HR, 0.47; 95% CI, 0.30–0.70; p < 0.001).

Moreover, the FOLFIRINOX group exhibited higher outcomes of both median OS and PFS. This might be attributed to the use of irinotecan, which is active against PDAC on its own and demonstrates synergistic action when administered prior to fluorouracil (123, 124). Furthermore, platinum-based oxaliplatin is more efficacious when combined with fluorouracil (125). According to a retrospective cohort research, a comparative analysis of randomized and controlled trials comparing FOLFIRINOX against GnP with regards to OS and PFS, in which 216 patients were randomly allocated either to the FOLFIRINOX (109 subjects), or the GnP group (107subjets), the FOLFIRINOX group had a better outcome versus the GnP group (median OS, 14-months (95% CI, 10–21) vs. 9 months (95% CI, 8–12, p, 0.008), even with adjustments for age, peritoneal carcinomatosis, extent of metastatic locations, liver metastases, and baseline CA19.9 level (HR, 0.67; p, 0.097) (126). This clinically relevant result cannot be attributed solely to FOLFIRINOX treatment because many patients within that group received GnP as second-line therapy (72.0% vs. 57.8%, respectively; p < 0.042), implying that the FOLFIRINOX group’s longer OS was due in part to the employment of GnP as a 2nd line therapy. FOLFIRINOX (FFX) followed by GnP (FFX–GnP) is the sequence that was found to be more practical (43.0%) than the reverse sequence (GnP–FFX) (12.8%; p 0.001) (**Table 1**). Furthermore, incorporating GnP as a sequential treatment following FOLFIRINOX failure was reported to enhance the median OS by 7.6 months, while the PFS median was 3.8 months. From the first dosage of FOLFIRINOX, the median OS was 14.2 months (95% CI, 10.6–15.1), with a cumulative median PFS of 9.3 months (95% CI, 7.5–12.4). The increase in median OS was offset by an increase in grades 3–4 adverse events, specifically hematology adverse reactions and peripheral sensory neurological toxicity. Moreover, a larger and more complex Korean retrospective research evaluated FOLFIRINOX with GnP as first-line therapy for metastatic PDAC (127). The findings favored GnP when compared to the chemotherapy (FOLFIRINOX), with a median average OS of 12.1 (95% CI, 10.7–) as well as 10.7 months (95% CI, 9.1–12.3), PFS between 8.0 and 8.4 months (p = 0.134), and 33.7% of objective response rates and 46.9% (p= 0.067), respectively. Nevertheless, these outcomes were not statistically relevant (127). Moreover, mFOLFIRINOX (modified regimen with the omission of 5FU bolus and attenuated Irinotecan dose) was shown to have comparable results to GnP with regards to median OS and PFS, as well as a favorable toxicity profile. According to Watanabe. K. et al., the GnP treatment outperformed mFOLFIRINOX in a real-world setting; the median OS extended to 14.0 months (95% CI, 12.2—not attained) versus 11.5 months (95% CI, 9.7–16.8), the PFS was 6.5 months (95% CI, 6.1–7.9) versus 5.7 months (95% CI, 3.4–7.1), and the 12-month survival rate was 44% versus 67% (p, 0.0006), respectively (128) (**Table 1**). A recent meta-analysis of twenty-two retrospective trials including 6351 patients found equivalent results between the GnP and mFOLFIRINOX with regards to median OS and PFS, as well as similar toxicity profiles (129). Based on the aforementioned findings, both regimens have been recommended as first-line treatments for patients with optimal performance status [ECOG PS of zero (0) or one (1)], whereas gemcitabine is recommended as first-line treatment for patients with poorer performance status [ECOG PS of higher than or equal to two] (2) (19, 99).

Each of the ASCO and NCCN guidelines currently advise sequencing fluoropyrimidine and gemcitabine-based therapies as second-line treatment for progressive advanced PDAC depending on first-line treatment and the degree of clinical response and effectiveness (130, 131).

For several years, investigators have explored new cytotoxic drug combinations as second-line treatments for highly advanced metastatic PDAC. For example, NAPOLI-1 was a multicenter, randomized, open-label trial that investigated the benefits of liposomal irinotecan (nal-IRI) plus 5FU plus leucovorin (LV) compared to liposomal Irinotecan only as second-line treatment in gemcitabine-resistant for metastatic PDAC patients. Regarding OS, the naI-IRI with 5FU and LV combination outperformed the 5FU combination with LV (OS, 6.2 vs 4.2 months; HR, 0.63; % CI, 0.47–0.85; p, 0.002) (**Table 1**). nal-IRI had no improvement over the 5FU combination with LV. Patients who were treated with nal-IRI with 5-FU and LV had a median PFS of 3.1 months vs 1.5 months in patients who received 5-FU with LV (HR, 0.57; 95% CI, 0.43–0.76; p 0.0001). It was also noted that, relatively younger age, higher performance status, absence of liver metastasis, lower levels of CA19–9, and a lesser overall neutrophil-lymphocyte ratio (5) were associated with better results in the nal-IRI with 5FU and LV group (110).

Based on the efficacy of nal-IRI in the second-line setting, the open-label, phase III NAPOLI-3 study aimed to assess the efficacy of nal-IRI with oxaliplatin and 5FU/LV (NALIFIROX) vs GnP as initial therapy for newly diagnosed metastatic PDAC. After a median follow-up of 16.1 months, the median OS was 11.1 months in the NALIFIROX arm vs 9.2 months in the GnP arm [HR 0.84 (95% CI 0.71–0.99); p = 0.04]. Gastrointestinal toxicity was more common with NALIFIROX whereas hematologic toxicity occurred more frequently with GnP (111). Questions regarding cost-effectiveness as well as efficacy compared to mFOLFIRINOX remain and complicate incorporation into clinical practice.

Targeted treatment has gained traction in recent years. The phase III POLO trial evaluated the efficacy and safety of olaparib as maintenance therapy following at least 16 weeks of first-line platinum-based chemotherapy with no evidence of progression in patients who had a germline BRCA1/BRCA2 mutation and metastatic PDAC. The median PFS was significantly prolonged in the olaparib arm (7.4 months vs. 3.8 months; HR, 0.53; 95% CI, 0.35 to 0.82; p=0.004). Interim OS results showed no statistical improvement with olaparib; however, subsequent treatment, including PARP inhibitors, could have confounded results (132). Given that KRAS mutations are present in 90% of PDAC cases, and KRAS G12C mutations arise in 1–2% of patients, leveraging KRAS G12C inhibitors presents a promising strategy (133). The phase 1–2 CodeBreaK 100 trial assessed the efficacy and safety of sotorasib in 38 patients with KRAS G12C–mutated metastatic PDAC. Notably, patients had received a median of 2 prior lines of therapy (range, 1–8). The objective response rate (ORR) was 21% (95% CI, 10–37), median PFS was 4 months (95% CI, 5–9.1), and median OS was 6.9 months (95% CI, 5.0 to 9.1). Grade 3 adverse events, mostly diarrhea and fatigue, occurred in 16% of patients and did not lead to drug discontinuation (134). Similarly, adagrasib, another KRAS G12C inhibitor, showed promising clinical activity in the phase 1–2 KRYSTAL-1 study (135). The study sought to evaluate the safety and efficacy of adagrasib in patients with solid tumors (excluding lung and colorectal cancer) and a confirmed KRAS G12C mutation. Out of the 64 enrolled patients, 21 patients had PDAC and had received a median of 2 previous lines of therapy. In that subset of patients, the ORR was 33%, median PFS was 5.4 months, and median OS was 8 months. Grade 3 adverse events, mostly fatigue and QT prolongation, occurred in 25.4% of patients and did not lead to drug discontinuation (135). While initial results with KRAS G12C inhibitors seem encouraging, larger, confirmatory trials demonstrating survival benefit are needed.

## 4 Precision oncology of PDAC

### 4.1 Pancreatic molecular profiling

Advances in sequencing technologies such as next generation sequencing (NGS) have allowed for the identification of molecular subtypes of PDAC with unique biomolecular traits and targetable features (136–138). Such classification may prove useful for drug development, diagnostic assessment, and ultimately creating individual treatment plans for patients with PDAC (138, 139).

Commonly altered pathways include AKT/mTOR (19%), cell cycle (11%), and DNA damage repair (15%) (136). Molecular-driven therapies have already demonstrated prognostic significance in terms of survival benefit as can be seen with immune check-point inhibitors for mismatch repair-deficient tumors and TRK inhibitors for tumors with ROS1, NTRK1, NTRK2, and NTRK3 gene fusions. Similarly, pancreatic tumors with BRAF mutations might receive help from therapy with RAF-MEK-targeted treatment (140, 141).

The Know Your Tumor (KYT) trial allowed pancreatic cancer patients to undergo multi-omics profiling and provided recommendations for molecular based clinical trials and personalized therapy (140, 141). Results of this trial showed that such uniquely tailored therapies that match the individuals altered genome improve 1-year survival benefits compared to those with unmatched therapies (140, 141).

Over the years, efforts in molecular tumor taxonomy have provided various proposals of resected pancreatic cancer subtypes. Collision et al. classified pancreatic cancer into 3 transcriptional subtypes including quasi-mesenchymal, classical, and exocrine-like that differ in prognosis and clinical response (142). Later, Moffitt et al. defined 2 tumor subtypes: basal-like, characterized by a worse prognosis, and classical. In a subsequent study, Bailey et al. conducted gene expression analysis and revealed four distinct subtypes: the squamous, pancreatic progenitor, immunogenic, and aberrantly differentiated endocrine exocrine (ADEX). Across all 3 classification systems, the basal-like, quasi-mesenchymal, and squamous subtypes were associated with mutations in genes involved in DNA methylation and worse prognosis. In contrast to the squamous subtype, the Bailey et al. pancreatic progenitor which also mirrored the Collisson et al. classical group was characterized by mechanisms involved in pancreatic endodermal differentiation and had better survival (142).

In the COMPASS trial, basal-like tumors showed less radiological response to first-line chemotherapy. Resistance to FOLFIRINOX, paclitaxel, and tyrosine kinase inhibitors was seen in basal-like tumors while the classical transcriptional subtype showed higher susceptibility for EGFR inhibition via erlotinib (143, 144). These findings support the necessity of utilizing molecular subtyping in therapeutic decisions of PDAC (143, 144).

Translation of NGS and molecular subtyping of PDAC may improve prognosis allowing precisely tailored oncologic therapy (140, 141). Resectable and borderline resectable pancreatic tumors might benefit from molecular-driven approaches due to a lower tumor load both before and after surgery in adjuvant therapy.

PDAC patients with non-molecularly targetable subtypes may be more freely considered for primary resection while those with molecularly targeted subtypes will benefit from optimal selection of neoadjuvant therapy and can avoid extensive resection, thus improving disease outcome. Therefore, it is necessary to integrate molecular subtyping into upcoming trials focused on resectable and/or borderline resectable PDAC.

### 4.2 Pancreatic head and pancreatic body/tail cancer treatment variation

**Figure 2:**
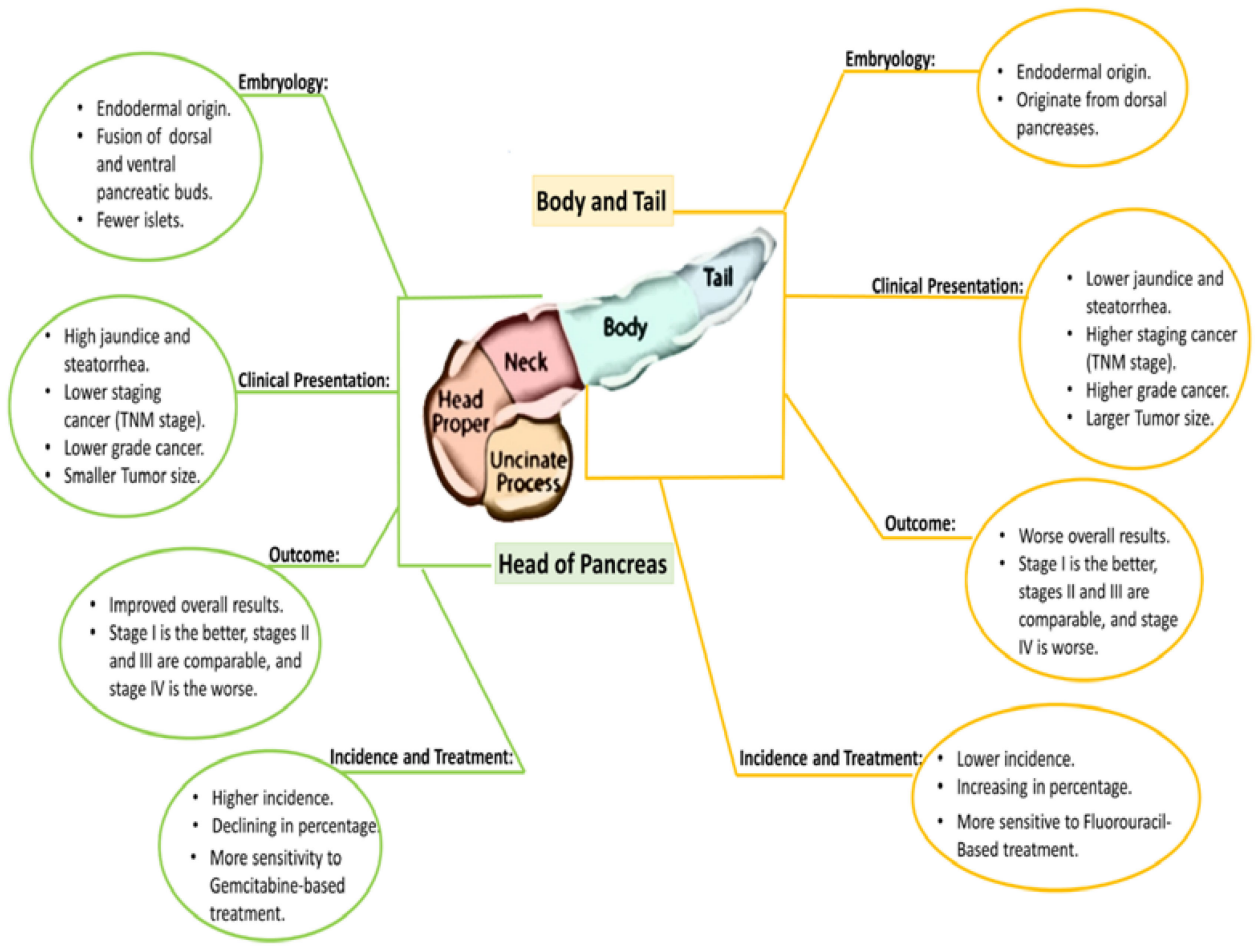
The differences of origin, clinical presentation, and treatment in the PDAC Head and tail.

The direct effect of PDAC’s anatomical location on survival rates has already been investigated in big national data-based investigations that have produced controversial conclusions (137–139, 145, 146); to determine if pancreatic head cancer (PHC) and pancreatic body/tail cancer (PBTC) (**Figure 2**) have differing OS, molecular signatures, and chemotherapy responses. Accordingly, a retrospective study (146), recorded between July 2016 and June 2020 included 101 patients, having full data, of which 66.34% (about 67 patients) were pancreatic head cancer and 33.66% (about 34 patients) were pancreatic body/tail cancer. Pancreatic head cancer was detected at a younger age (61.49 compared to 68.97, P = 0.010), an earlier stage (P = 0.006), and with surgical resection (P = 0.025) Not includingTP53 mutations (37.0% in PHC against 70.0% for pancreatic body/tail cancer, P = 0.03), there were no big variations across other mutations and pathways investigated (146). In the general population as well as in subgroups based on surgical resection status or stages, there was no significant difference in OS between pancreatic head cancer and pancreatic body/tail cancer (P = 0.636). Regarding chemotherapy response, chemotherapy treatments (FOLFIRINOX-based against Gemcitabine-based) did not affect the cancer-free period in those who had surgical resection, whether in pancreatic head cancer (P = 0.546) or pancreatic body/tail cancer (P = 0.654) or even the entire duration of response to the first line of palliative therapy among those with advanced stages in pancreatic head cancer (P = 0.915) or pancreatic body/tail cancer patients (P = 0.524). Although both pancreatic head cancer and pancreatic body/tail cancer have similarly unfavorable outcomes and responses to chemotherapy (**Figure 2**), the differences in their presentations and molecular profiles show that they are different illnesses. Individualization of treatment requires the use of molecular profiling technology for further development of targeted therapy. In the same direction, Abdelrahim et al. reported an excellent study that showed the pancreatic head of adenocarcinoma has a different tumor microenvironment than the body and tail of the pancreatic adenocarcinoma which introduces the pancreatic head of adenocarcinoma as a potential responder to immunotherapy (147).

## 5 Conclusion

Imaging is crucial in PDAC diagnosis, monitoring, resectability appraisal, and response evaluation. The advancement of medical technologies has grown in imaging as well as the utilization of ctDNA as a tumor marker in PDAC. Several challenges such as predicting the prognosis of resectable PDAC, assessment of therapy response, and mitigating the poor prognosis associated with unresectable PDAC are yet to be resolved. Surgery remains the sole curative option for PDAC. The cornerstone of PDAC treatment is adjuvant and neoadjuvant chemotherapy. However, a slew of new targeted/immunomodulatory therapies, as well as cytotoxic medications are easing the burden, with advancements in PDAC management likely in the upcoming decade. Furthermore, examining genetic abnormalities in PDAC is regarded as an essential step in formulating a well-tailored and individualized therapeutic plan, especially with breakthroughs in advanced gene sequencing.

## Author contributions

DB: Conceptualization, Data curation, Investigation, Methodology, Software, Visualization, Writing – original draft, Writing – review & editing. EA-N: Conceptualization, Data curation, Investigation, Resources, Software, Writing – original draft, Writing – review & editing. AE: Conceptualization, Data curation, Formal analysis, Funding acquisition, Investigation, Methodology, Project administration, Resources, Software, Supervision, Validation, Visualization, Writing – original draft, Writing – review & editing. SA: Conceptualization, Writing – original draft, Writing – review & editing. KA: Writing – original draft, Writing – review & editing. AdA: Writing – original draft, Writing – review & editing. GU: Writing – original draft, Writing – review & editing. HR: Writing – original draft, Writing – review & editing. AlA: Writing – original draft, Writing – review & editing. A-EA: Writing – original draft, Writing – review & editing. MA: Conceptualization, Data curation, Formal analysis, Funding acquisition, Investigation, Methodology, Project administration, Resources, Software, Supervision, Validation, Visualization, Writing – original draft, Writing – review & editing.
